# A comparative study revealed first insights into the diversity and metabolisms of the microbial communities in the sediments of Pacmanus and Desmos hydrothermal fields

**DOI:** 10.1371/journal.pone.0181048

**Published:** 2017-07-12

**Authors:** Hai-liang Wang, Jian Zhang, Qing-lei Sun, Chao Lian, Li Sun

**Affiliations:** 1 Key Laboratory of Experimental Marine Biology, Institute of Oceanology, Chinese Academy of Sciences, Qingdao, China; 2 Laboratory for Marine Biology and Biotechnology, Qingdao National Laboratory for Marine Science and Technology, Qingdao, China; 3 University of Chinese Academy of Sciences, Beijing, China; 4 Key Laboratory of Marine Geology and Environment, Institute of Oceanology, Chinese Academy of Sciences, Qingdao, China; National Cheng Kung University, TAIWAN

## Abstract

Currently, little is known about the microbial diversity in the sediments of Pacmanus and Desmos hydrothermal fields in Manus Basin. In this study, Illumina-based sequencing of 16S rRNA gene amplicons and metagenomic analysis were conducted to investigate the microbial populations and metabolic profiles in the sediments from four different regions in Pacmanus and Desmos hydrothermal fields. It was found that *Gammaproteobacteria* and Thaumarchaeota were the most abundant bacterial and archaeal populations, respectively. The autotrophic prokaryotes in the four communities probably fixed CO_2_ via four major pathways, i.e. Calvin-Benson-Bassham cycle, reductive acetyl-CoA cycle, rTCA cycle, and 3-hydroxypropionate/4-hydroxybutyrate cycle. Ammonia-oxidizing Thaumarchaeota, nitrifiers, denitrifiers, and sulfur oxidizers belonging to the subgroups of Proteobacteria (e.g., alpha, beta, gamma, and epsilon), *Nitrospira*, and *Nitrospina*, and sulfate-reducing *Desulfobacterales* likely played critical roles in nitrogen and sulfur cycling, in which ammonia, sulfur compounds, and hydrogen could be utilized as potential energy sources. These findings revealed new insights into the operational mechanism of the microbial communities associated with Pacmanus and Desmos hydrothermal fields.

## Introduction

Manus Basin is located behind the New Britain arc-trench system [1]. Pacmanus, Desmos, and Susu Knolls are the three major hydrothermal fields in the eastern part of the basin [2,3]. The Pacmanus field in Pual Ridge was discovered in 1991, which consists of five discrete high-temperature hydrothermal sites (Roger’s Ruins, Roman Ruins, Satanic Mills, Tsukushi, and Fenway), a low-temperature diffuse vent site (Snowcap), and four new vent sites (Mimosa, Solwara 6, 7, and 8) [2–6]. The Snowcap area is heavily sedimented with hydrothermal precipitate/volcaniclastic debris and covered by bacterial mats [5,7]. In Fenway, a large black smoker chimney has been found to discharge the highest temperature fluids (358°C) observed at the Pacmanus field [5]. Solwara 8 is located southeast of Fenway and hosts black smoker chimneys releasing high-temperature fluids [6].

Desmos was discovered in 1990 and is located 23 km further east of Pacmanus [2,8]. In this vent field, milky-white fluids with low pH and high concentrations of H_2_S and SO_4_^2–^ discharge directly through altered volcanic breccia and hydrothermal sediments composed of abundant native sulfur and anhydrite [1]. The pH values range from 1.5 to 3.0 in low temperature fluids (88~120°C) in Desmos field and in high temperature fluids (241~358°C) in Pacmanus field [1,5,9]. The remarkable fluid acidity has been suggested to be due to the magmatic gases CO_2_, SO_2_, and HF [1,5,10]. In addition, the acidic hydrothermal fluid likely contributes to the high aluminum concentrations in the hydrothermal plumes collected from Desmos [8]. Studies on mineral composition and hydrothermal fluid chemistry indicate magmatic contributions to the hydrothermal activities in Pacmanus and Desmos [1,5,8,10,11].

To date, two studies on the microbes in the hydrothermal fields of Manus Basin have been documented [12,13]. In one of these studies, the distribution of microorganisms in the subsurfaces of a hydrothermal vent in Papua New Guinea was investigated by using subvent rock core samples, in which microbial cells were detected at depths of less than 99.4 m below the seafloor [12]. In another study, the distribution of archaea in a black smoker chimney structure in the Pacmanus site near Papua New Guinea was determined [13]. However, nothing is known about the microbial populations in the sediments from Pacmanus and Desmos hydrothermal fields.

The primary goal of the present study was to investigate the composition, diversity, and metabolism of the microbial communities inhabiting the sediments located at different regions in Pacmanus and Desmos hydrothermal fields. To achieve this purpose, we employed Illumina HiSeq-based sequencing, a technique frequently used to study microbial diversity in various environments [14–16], and metagenomic approaches. The results of this study will facilitate our understanding of microbial survival strategies in deep-sea hydrothermal systems.

## Materials and methods

### Sample collection

The samples used in this study were collected in June, 2015 during the cruise conducted by the scientific research vessel KEXUE in Manus Basin. The cruise was approved by Chinese Ministry of Foreign Affairs and with permit from the relevant country. The sediment samples approximately 100 m from active vents in Pacmanus and Desmos hydrothermal fields were collected by push-cores on ROV equipped on KEXUE vessel. Samples PR1 (151°40'11.608''E, 3°43'43.216''S; 1680 m) and PR4 (151°40'38.477''E, 3°44'02.482''S; 1853 m) were from Pacmanus; samples DR7 (151°52'49.685''E, 3°42'46.446''S; 2003 m) and DR11 (151°51'50.212''E, 3°40'45.187''S; 1906 m) were from Desmos. The temperature and concentrations of CO_2_ and CH_4_ of the sampling sites were *in situ* detected (Table 1). After sampling, the superficial 3–5 cm layer of each sediment core was removed on board before sediment division with sterile operation. Surface sediments (upper 10 cm), which were used in the study, were contained in aseptic sampling bags (Haibo, Qingdao, China). The samples were stored at -80°C and kept on dry ice during transportation.

**Table 1:** Information of the sampling sites in this study.

	PR1	PR4	DR7	DR11
**Temperature (°C)**	1.03	1.02	1.01	1.01
**Depth (m)**	1680	1853	2003	1906
**CO**_**2**_ **(ppm)**	636.08	571	500	494
**Location**	151°40'11.608''E, 3°43'43.216''S	151°40'38.477''E, 3°44'02.482''S	151°52'49.685''E, 3°42'46.446''S	151°51'50.212''E, 3°40'45.187''S
	Pacmanus	Pacmanus	Desmos	Desmos

### Chemical analysis

Chemical compositions of the samples were analyzed by the Research Center of Analysis and Measurement, Institute of Oceanology, Chinese Academy of Sciences. The details of sample processing have been described previously [17,18]. The concentrations of total organic carbon (TOC), total nitrogen (TN) and total sulfur (TS) were determined using an elemental analyzer (Elementar, Germany). Metal elements were analyzed with an inductively coupled plasma optical emission spectroscopy (Perkin Elmer, USA) using a strong acid digestion method [19].

### DNA extraction

Approximately 0.3 g of each sample was used for total DNA extraction using TIANamp Soil DNA Kit (Tiangen, Beijing, China) following the manufacturer’s protocol. DNA was qualified using NanoPhotometer spectrophotometer (IMPLEN, CA, USA) and Qubit dsDNA Assay Kit.

### Sequencing and analysis of 16S rRNA gene amplicons

Total DNA was used as the template for PCR amplification of 16S rRNA gene using 6-nt barcoded primers 341F (5'-CCTAYGGGRBGCASCAG-3') and 806R (5'-GGACTACNNGGGTATCTAAT-3') which target hypervariable regions V3 to V4 of 16S rRNA gene [20]. Details of amplification have been described by Zhang et al. [18]. The mixture of purified 16S rRNA gene amplicons was used for library preparation. The library was constructed using TruSeq^®^ DNA PCR-Free Sample Preparation Kit (Illumina, USA) following manufacturer’s recommendations. The quality of the library was assessed with Qubit 2.0 Fluorometer (Thermo Scientific, USA) and Agilent Bioanalyzer 2100 system. The library was sequenced on an Illumina HiSeq 2500 platform in Novogene (Tianjin, China). Paired-end (PE) raw reads were generated and subsequently deposited in NCBI Sequence Read Archive (SRA) (http://www.ncbi.nlm.nih.gov/Traces/sra/) database under accession SRP079171.

Sequence assembly and quality control processing of the raw reads were done as reported previously [18]. The raw reads without barcodes and 16S rRNA gene primers were assembled to raw tags using FLASH [21]. Low-quality tags were then filtered with QIIME V1.7.0 [22]. Chimera sequences were identified and excluded using UCHIME version 7.0.1001 [23] after alignment with 16S reference database available at http://drive5.com/uchime/gold.fa. Quality-filtered tags were clustered into operational taxonomic units (OTUs) at 97% similarity using UPARSE version 7.0.1001 [24]. Taxonomic assignment of representative OTUs was performed using Blast program (e-value cutoff of 1 × 10^−5^) against the RDP database (v10) [25]. Before alpha diversity calculations, the read numbers were normalized to that of the sample (DR11) with the smallest read number (28772). Indexes (Shannon and Chao 1) of microbial diversity and species richness were calculated with QIIME.

### Metagenome sequencing and analysis

One microgram of total DNA per sample was fragmented into ~300 bp by sonication and used to construct metagenomic library using NEBNext^®^ Ultra^TM^ DNA Library Prep Kit (NEB, USA) as recommended by the manufacturer. After quality control processing, the library was sequenced on an Illumina HiSeq 4000 platform. PE raw reads were generated after sequencing, and clean data were extracted from the raw reads following the removal of adaptor fragments and low quality reads. High-quality clean reads were assembled using IDBA_UD [26] into scaffolds. Scaftigs were generated after split of “N” sequences within the scaffolds, and scaftigs shorter than 500 bp were filtered out. USEARCH version 7.0.1001 [27] was used to select unique scaftigs with 100% identity from all metagenomic datasets. The abundance of unique scaftigs in every metagenome was calculated, after mapping the corresponding clean data to the unique scaftigs using SoapAligner (version 2.21) [28]. The open reading frames (ORFs) contained in the unique scaftigs were predicted by MetaGeneMark [29]. The predicted ORFs were clustered using CD-HIT (version 4.5.8) with ≥ 95% identity and ≥ 90% overlap [30,31], and the representatives were selected subsequently and used for functional annotation. For functional annotation, the deduced amino acid sequences of the ORFs were searched against Kyoto Encyclopedia of Genes and Genomes (KEGG) database (Release 59.0) with the BLASTP program (e-value ≤ 1 × 10^−5^) [32]. All metagenomic datasets have been deposited in the NCBI SRA database under the accession number SRP079171.

### Phylogenetic analysis

Query amino acid sequences were searched against the non-redundant protein database in NCBI (https://www.ncbi.nlm.nih.gov/) using BLASTP program, and the chosen closely related reference sequences were either share an identity of >50% and a query cover of >90% with the query sequence or rank top 10 in the Blast Hits (40% < identity <50% and query cover >80%). Other subject sequences as in-groups which had relatively mediate or low identity with query sequences were also obtained from the Blast Hits. Multiple sequence alignments were completed with the CLUSTAL_X program [33]. Phylogenetic trees were constructed with the Neighbor-Joining algorithm under the parameter settings of p-distance substitution model and pairwise deletion for gap treatment in MEGA 6 [34], and bootstrap values were based on percentages of 1000 replicates.

## Results

### Chemical characteristics of the sediment samples

Four sediment samples, two from Pacmanus hydrothermal field (named PR1 and PR4) and two from Desmos hydrothermal field (named DR7 and DR11), were used in this study (Table 1). The chemical compositions of the four samples are summarized in Table 2. The contents of TOC and TN were similar in the four sediments, while the content of TS was approximately 3 to 8 folds higher in DR7 (0.205%) and DR11 (0.282%) than in PR1 (0.036%) and PR4 (0.069%). In all samples, the concentrations of Al, Fe, Ca, Mg, and Mn (over 11000 μg/g) were much higher than that of Ba, Cu, Zn, As, Pb, and Co (19 to 878 μg/g) (Table 2). Aluminum was the most abundant metal ion, ranging from 62510 μg/g to 75430μg/g in the four samples, while cobalt was the least abundant, ranging from 19.5 μg/g to 30 μg/g. For heavy metals, Cu, Zn, and Pb were most abundant in PR1, with concentrations being 878.1 μg/g, 352.0 μg/g, and 129.4 μg/g, respectively (Table 2).

**Table 2:** Chemical and metal components of the sediment samples in this study.

Sample	TOC^a^ (%)	TN^b^ (%)	TS^c^ (%)	Metal ion (μg/g)										
				Al	Fe	Ca	Mg	Mn	Ba	Cu	Zn	As	Pb	Co
**PR1**	0.43	0.06	0.036	64710	43380	34710	11800	6443	763.3	878.1	352.0	180.4	129.4	19. 5
**PR4**	0.83	0.11	0.069	65040	51890	49380	15060	7535	397.6	277.7	231.5	145.6	62.4	25.3
**DR7**	0.97	0.12	0.205	75430	57750	44110	18700	11750	281.6	125.4	135.3	178.4	24.5	26.5
**DR11**	0.71	0.09	0.282	62510	62920	45040	22510	3914	262.8	197.9	155.4	178.2	32.1	30.0

^a^TOC, total organic carbon

^b^TN, total nitrogen

^c^TS, total sulfur.

### Microbial diversity based on 16S rRNA gene sequence

Following sequencing 16S rRNA gene amplicons, 63071, 74331, 66522, and 76595 raw PE reads were retrieved from PR1, PR4, DR7, and DR11 respectively (S1 Table). A total of 186041 high-quality reads (“effective tags”) from the four samples were used for further analysis. The effective tags clustered into 1169, 1636, 1710, and 1617 OTUs in PR1, PR4, DR7, and DR11 respectively (S1 Table). There was no clear difference in Chao1 richness or Shannon diversity among the four samples (S1 Table).

**Fig 1:**
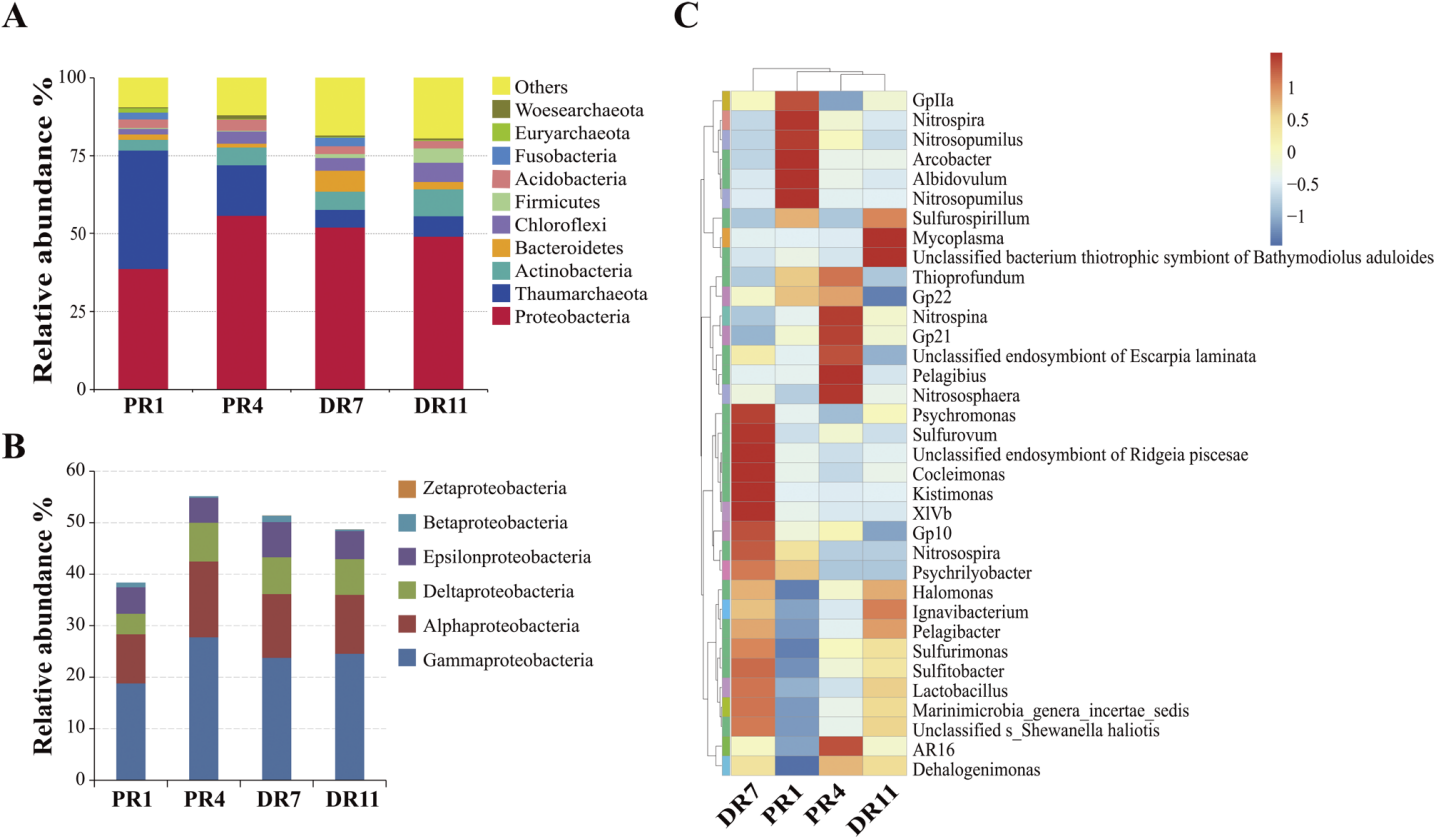
Composition of the microbial communities in the four samples. (A) The 10 dominant phyla are shown with their relative abundances; the remaining phyla are indicated as “Others”. (B) The relative abundance of Proteobacteria phylum. (C) Heat map of the microbial compositions of the four samples at genus level, drawn based on top 35 abundant genera. The relative values of the genera are indicated by colors of different intensities with legend at the right of the figure. The genera are indicated by different colors (vertical clustering) according to which phylum they belong to.

Proteobacteria was the dominant bacterial phylum (relative abundance ranging from 38.7% to 55.8%) in all samples, and Thaumarchaeota was the dominant archaeal phylum (Fig 1A). The abundances of Thaumarchaeota were 38.0%, 16.2%, 6.5%, and 5.6% in PR1, PR4, DR11, and DR7, respectively. Other abundant phyla included Actinobacteria, Bacteroidetes, Chloroflexi, Firmicutes, Acidobacteria, Fusobacteria, Elikeuryarchaeota, and Woesearchaeota (Fig 1A). Within Proteobacteria, *Gamma-* and *Alphaproteobacteria* were the dominant classes, accounting for ~73% of the Proteobacteria community in the four samples, which were followed in abundance by *Delta-* and *Epsilonproteobacteria* (Fig 1B). The relative abundance of *Deltaproteobacteria* in PR1 (4.0%) was apparently lower than that in PR4 (7.5%), DR7 (7.1%), and DR11 (6.9%). The relative abundances of *Epsilonproteobacteria* in the four samples ranged from 4.8% to 6.8%.

At genus level, the most abundant genera in PR1, PR4, DR7, and DR11 were *Nitrosopumilus* (37.9%), *Nitrosopumilus* (15.8%), unclassified endosymbiont of *Escarpia laminate* (6.2%), and unclassified bacterium thiotrophic symbiont of *Bathymodiolus aduloides* (12.2%), respectively. The relative abundances of *Nitrosopumilus* were 5.5% and 6.4% in DR7 and DR11, respectively. *Sulfurovum* was also an abundant genus, accounting for 3.7%, 4.2%, 5.8%, and 3.7% in PR1, PR4, DR7, and DR11, respectively. A genus-level heat map was drawn based on the top 35 most abundant genera (Fig 1C).

**Fig 2:**
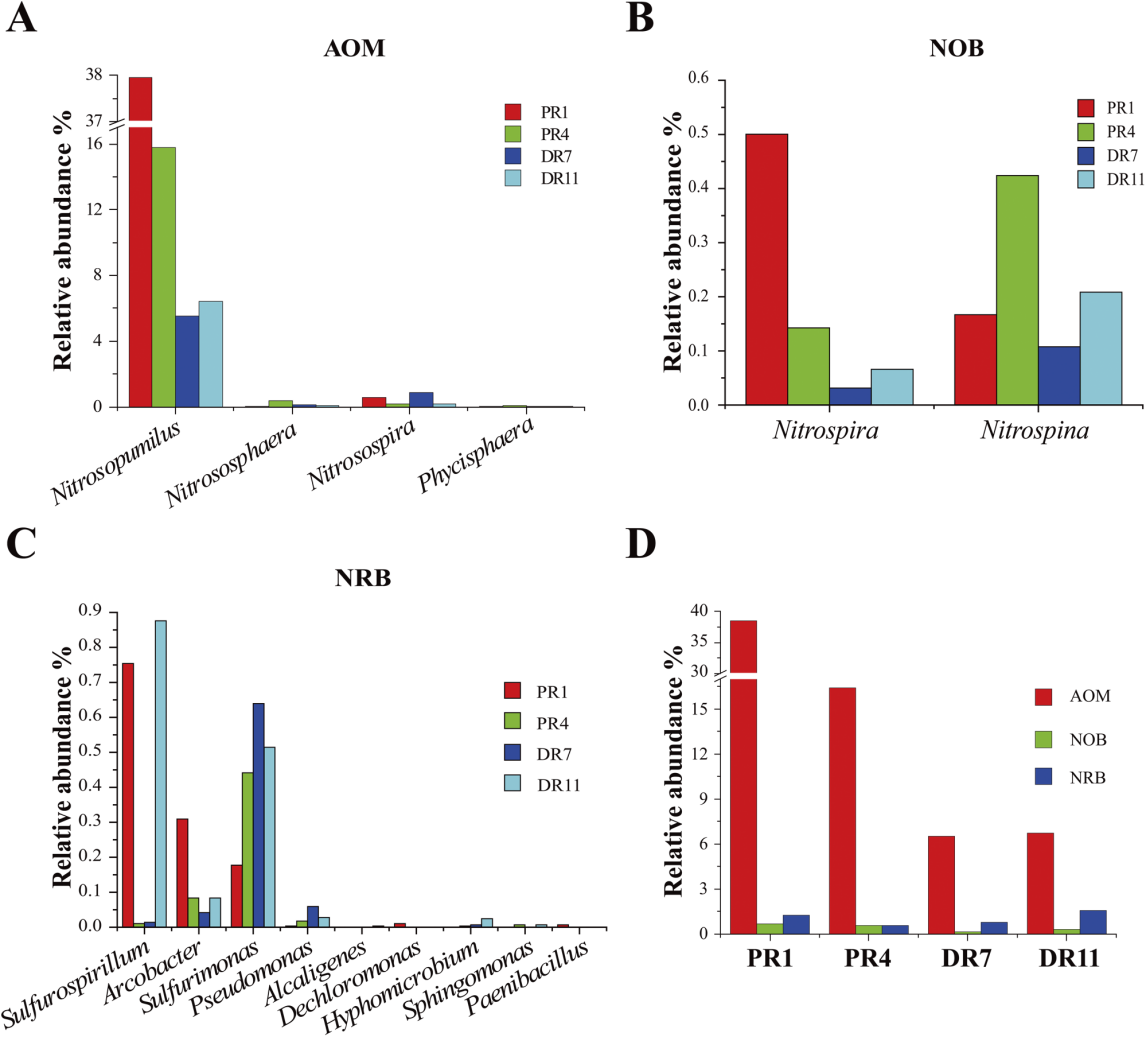
Microorganisms potentially involved in nitrogen metabolism. (A) Ammonia-oxidizing microorganisms (AOM). (B) Nitrite-oxidizing bacteria (NOB). (C) Nitrate-reducing bacteria (NRB). (D) Relative abundance of AOM, NOB, and NRB in every sample.

In the four samples, there existed microorganisms that potentially participated in oxidation of ammonia and nitrite and reduction of nitrate. Of the OTUs associated with ammonia-oxidizing microorganisms (AOM), most were related to the archaeal genus *Nitrosopumilus*, and the rest were related to the archaeal genus *Nitrososphaera* and the bacterial genera *Nitrosopira* and *Phycisphaera* (Fig 2A). Potential nitrite-oxidizing bacterial (NOB) genera were associated with *Nitrospira* and *Nitrospina* (Fig 2B). The relative abundances of OTUs associated with nitrate-reducing bacteria (NRB) varied in the samples (Fig 2C). Among the potential nitrate reducers, *Sulfurospirillum* was dominant in PR1 and DR11, while *Sulfurimonas* was dominant in PR4, DR7, and DR11. *Arcobacter* was next to *Sulfurospirillum* and *Sulfurimonas* in abundance. These three genera belong to *Epsilonproteobacteria*. The relative abundance of AOM was strikingly higher than that of NOB and NRB in each sample. AOM was relatively more abundant in the Pacmanus samples PR1 (38.56%) and PR4 (16.41%) than in the Desmos amples DR7 (6.53%) and DR11 (6.71%) (Fig 2D).

**Fig 3:**
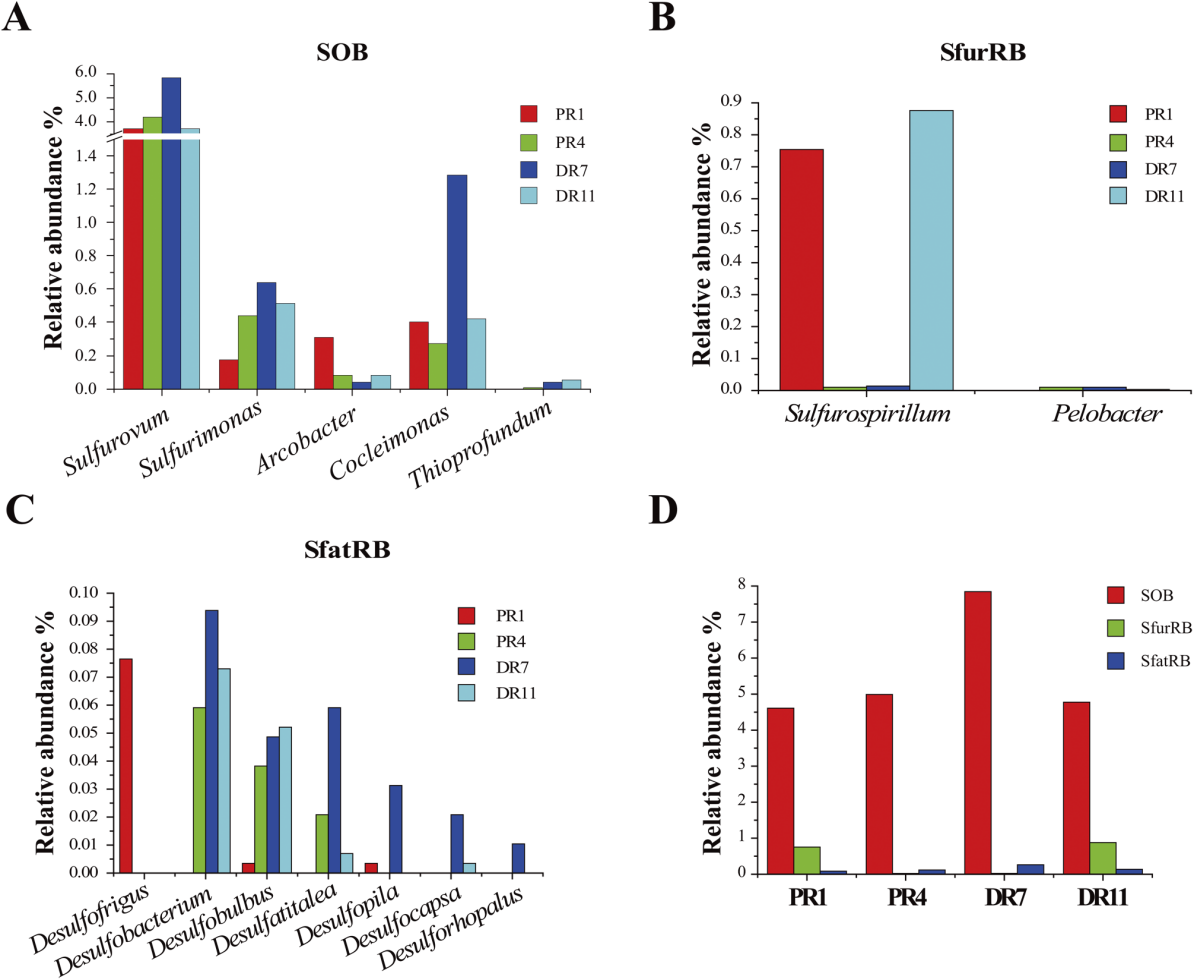
Bacteria involved in sulfur metabolism. (A) Sulfur-oxidizing bacteria (SOB). (B) Sulfur-reducing bacteria (SfurRB). (C) Sulfate-reducing bacteria (SfatRB). (D) Relative abundances of SOB, SfurRB, and SfatRB in the four samples.

Bacteria potentially playing important roles in sulfur metabolism were also detected. At the genus level, for sulfur-oxidizing bacteria (SOB), *Sulfurovum* was most abundant in the four samples (3.7% to 5.8%), which was followed in abundance by *Sulfurimonas*, *Cocleimonas*, *Arcobacter*, and *Thioprofundum* (Fig 3A). For sulfur-reducing bacteria (SfurRB), *Sulfurospirillum* and *Pelobacter* were detected. *Sulfurospirillum* was rare in PR4 (0.01%) and DR7 (0.01%) but relatively abundant in PR1 (0.75%) and DR11 (0.88%); *Pelobacter* was absent in PR1 but present in PR4 (0.01%), DR7 (0.01%), and DR11 (0.004%) (Fig 3B). For sulfate-reducing bacteria (SfatRB), they included, in the order of decreasing abundance, *Desulfofrigus*, *Desulfobacterium*, *Desulfobulbus Desulfatitalea*, *Desulfopila*, *Desulfocapsa*, and *Desulforhopalus* (Fig 3C). *Desulfofrigus* was only present in PR1 (0.076%), while *Desulfobacterium* was present in PR4 (0.059%), DR7 (0.094%), and DR11 (0.073%) but not in PR1. Comparative analysis indicated that SOB dominated in all samples, with relative abundance ranging from 4.6% to 7.8%, while the abundances of SfurRB and SfatRB ranged from 0.02% to 0.88% and 0.08% to 0.26%, respectively.

### Metabolic profiles of the microbial communities based on metagenomic analysis

Using an Illumina HiSeq platform, we obtained 52.9 G metagenomic raw reads (~13.2 G per sample) from the four samples. S2 Table summarizes the sequencing information. In average, 350335 high-quality scaftigs longer than 500 bp were obtained from each sample and used for further analysis.

#### (i) Genes involved in carbon metabolism

In the dark world of deep-sea hydrothermal fields, CO_2_ fixation pathways, including the Calvin-Benson-Bassham (CBB) cycle, reductive tricarboxylic acid (rTCA) cycle, reductive acetyl-CoA or Wood-Ljungdahl (WL) pathway, 3-hydroxypropionate (3-HP) cycle, dicarboxylate/4-hydroxybutyrate (DC/4-HB) cycle and 3-hydroxypropionate/4-hydroxybutyrate (3-HP/4-HB) cycle, are essential for microorganisms [35,36]. In our study, we found that in all four metagenomes, the CBB cycle and WL pathway were complete, while the 3-HP and 3-HP/4-HB cycles were incomplete. By contrast, complete rTCA cycle was present in PR1, PR4, and DR7, while complete DC/4-HB cycle was only present in DR7 and DR11. However, most key enzyme-coding genes in the autotrophic CO_2_ fixation pathways were detected in the four metagenomic datasets (Table 3). The relative abundance of *rbcLS* gene, which encodes the key enzyme ribulose-1,5-bisphosphate carboxylase of the CBB cycle, was higher in PR1 (0.0149%) than in PR4 (0.0076%), DR7 (0.0071%), and DR11 (0.0089%). Phylogenetic analysis based on RbcL sequences indicated that the RbcL in our samples was likely derived from uncharacterized taxa affiliated to the orders of *Chromatiales*, *Rhizobiales*, *Planctomycetales*, *Cytophagales*, *Oceanospirillales*, and *Sedimenticola*/*Thiotrichales* (S1 Fig). In contrast to *rbcLS*, the relative abundance of *cdh*, which encodes the key enzyme acetyl-CoA decarbonylase/synthase in WL pathway, was higher in PR4 (0.0121%), DR7 (0.0252%), and DR11 (0.0214%) than in PR1 (0.0002%). The deduced CdhA sequences were similar to those from the archaeal taxa Candidatus Bathyarchaeota (75% similarity) and Candidatus Thorarchaeota (84% similarity) (S2 Fig). Unlike *rbcL* and *cdh*, the *acl* gene encoding the key enzyme ATP-citrate lyase in rTCA cycle was absent or very low in abundance in the four metagenomic datasets (Table 3). Phylogenetic analysis based on AclA sequences showed that the AclA in our samples was likely associated with uncharacterized *Nitrospira* and *Thermoplasmatales* (S3 Fig). For the gene encoding the key enzyme 4-hydroxybutyryl-CoA dehydratase (*abfD*) in DC/4-HB and 3-HP/4-HB cycles, its abundances in the four samples were comparable (Table 3). These AbfD sequences were assigned to uncharacterized taxa belonging to the archaeal genus *Nitrosopumilus* and the bacterial genera *Plesiocystis*, unclassified *Dehalococcoidia*, and *Syntrophobacter* (S4 Fig).

**Table 3:** Relative abundances of the genes encoding key enzymes of CO_2_ fixation.

Pathway	Key enzyme	EC No.	Gene	Relative abundance (%)			
				PR1	PR4	DR7	DR11
**CBB cycle**^a^	Ribulose-bisphosphate carboxylase	4.1.1.39	*rbcLS*	0.0149	0.0076	0.0071	0.0089
**WL pathway**^b^	Acetyl-CoA decarbonylase/synthase	1.2.7.4	*cdh*	0.0002	0.0121	0.0252	0.0214
**rTCA cycle**^c^	ATP-citrate lyase	2.3.3.8	*aclAB*	0.0010	0.0002	0.0002	0.0000
**3-HP cycle**^d^	Malyl-CoA lyase	4.1.3.24	*mcl*	0.0048	0.0050	0.0037	0.0037
**DC/4-HB cycle**^e^ **or 3-HP/4-HB cycle**^f^	4-hydroxybutyryl-CoA dehydratase	4.2.1.120	*abfD*	0.0159	0.0124	0.0110	0.0125

^a^CBB cycle, Calvin-Benson-Bassham cycle.

^b^WL pathway, Wood-Ljungdahl pathway or reductive acetyl-CoA pathway.

^c^rTCA cycle, reductive tricarboxylic acid cycle.

^d^3-HP cycle, 3-hydroxypropionate cycle.

^e^DC/4-HB cycle, dicarboxylate/4-hydroxybutyrate cycle.

^f^3-HP/4-HB cycle, 3-hydroxypropionate/4-hydroxybutyrate cycle.

#### (ii) Genes involved in nitrogen metabolism

**Fig 4:**
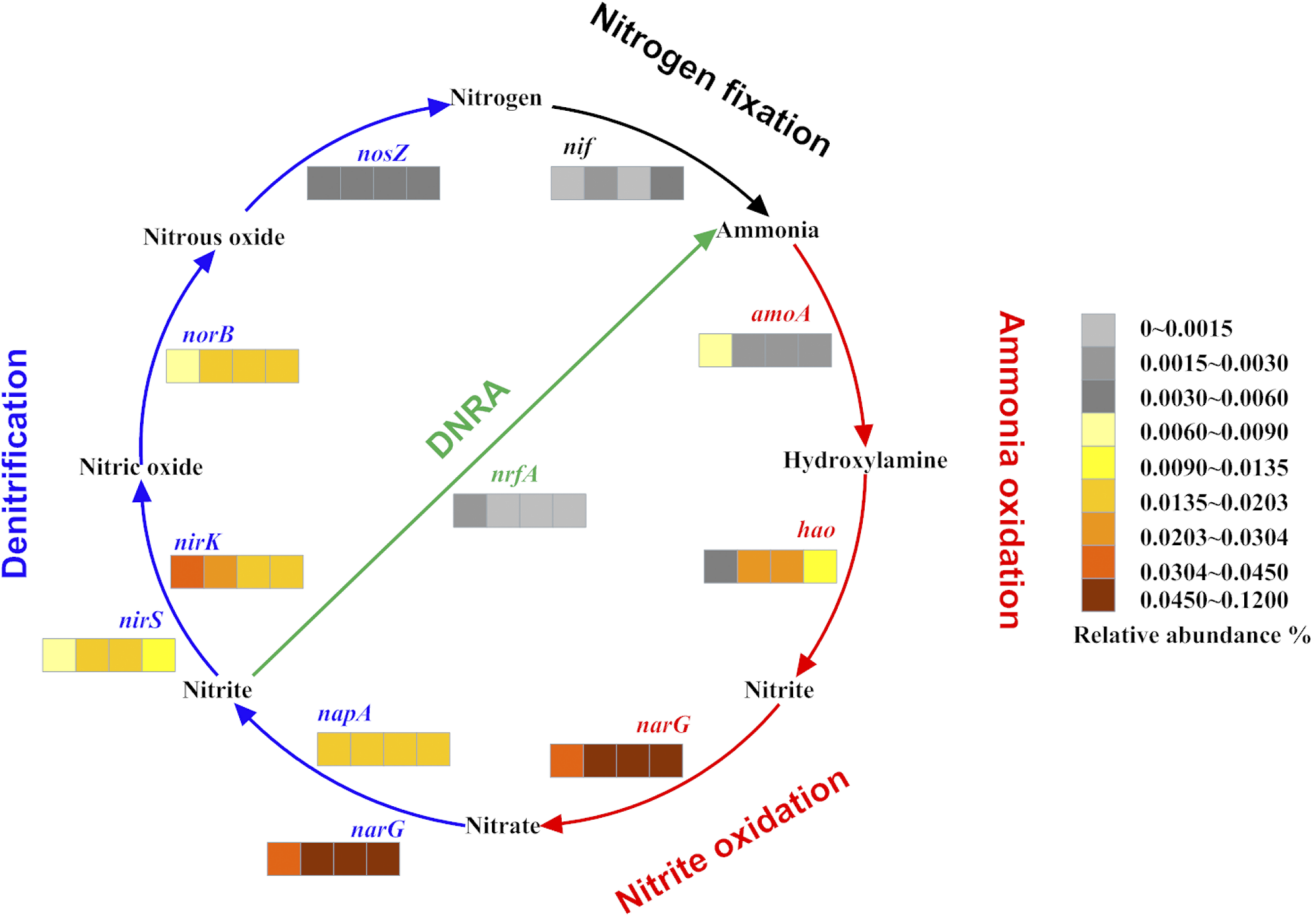
Relative abundance of the genes encoding key enzymes for nitrification, denitrification, and DNRA. Pathways and genes are indicated with different colors. The relative abundances of the gene in the four samples are shown in the nearby color bar, in which the four segments from left to right represent PR1, PR4, DR7, and DR11. DNRA, dissimilatory nitrate reduction to ammonium; *amoA*, encoding ammonia monooxygenase subunit A; *hao*, encoding hydroxylamine dehydrogenase; *narG*, encoding cytoplasmic nitrate reductase alpha subunit; *napA*, encoding periplasmic nitrate reductase; *nirK*, encoding copper-containing nitrite reductase; *nirS*, encoding cytochrome *cd*1 nitrite reductase; *norB*, encoding nitric oxide reductase; *nosZ*, encoding nitrous oxide reductase; *nif*, encoding nitrogenase; *nrfA*, encoding ammonia-forming dissimilatory nitrite reductase.

To examine the nitrogen metabolism pathways, we determined the relative abundances of the genes encoding the key enzymes for nitrification, denitrification, and dissimilatory nitrate reduction to ammonium (DNRA) (Fig 4). The initial reaction in denitrification is catalyzed by nitrate reductase. Phylogenetic analysis based on the sequences of respiratory nitrate reductase beta subunit (NarH) and periplasmic nitrate reductase small subunit (NapB) indicated that these sequences were likely associated with uncharacterized taxa belonging to the orders of *Rhizobiales*, *Burkholderiales*, *Alteromonadales*, Candidatus *Methylomirabilis*, *Myxococcales*, *Nitrospirales*, *Nitrospinales*, Candidatus *Brocadiales*, *Cellvibrionales*, *Pseudomonadales*, *Campylobacterales*, *Desulfobacterales*, and Candidatus Rokubacteria (S5 Fig). Nitrification includes two processes of nitrite formation via ammonia oxidation and nitrite oxidation to nitrate. For ammonia oxidation, two enzymes, ammonia monooxygenase (AMO) and hydroxylamine dehydrogenase (HAO) encoded by *amoA* and *hao*, respectively, catalyze the conversion of ammonia to nitrite. In our study, *amoA* exhibited low abundances (0.0017% to 0.0071%) in all samples, while *hao* exhibited relatively high abundances in PR4 (0.0234%), DR7 (0.0222%), and DR11 (0.0135%) but a low abundance in PR1 (0.0037%). Phylogenetic analysis showed that AmoA was associated with the uncharacterized genera *Nitrosopumilus* and *Nitrosospira* (S6 Fig). With respect to DNRA, the gene *nrfA*, which encodes the key enzyme ammonia-forming dissimilatory nitrite reductase of DNRA, was present in low abundance (~0.0009%) in each metagenomic dataset.

#### (iii) Genes involved in sulfur metabolism

**Fig 5:**
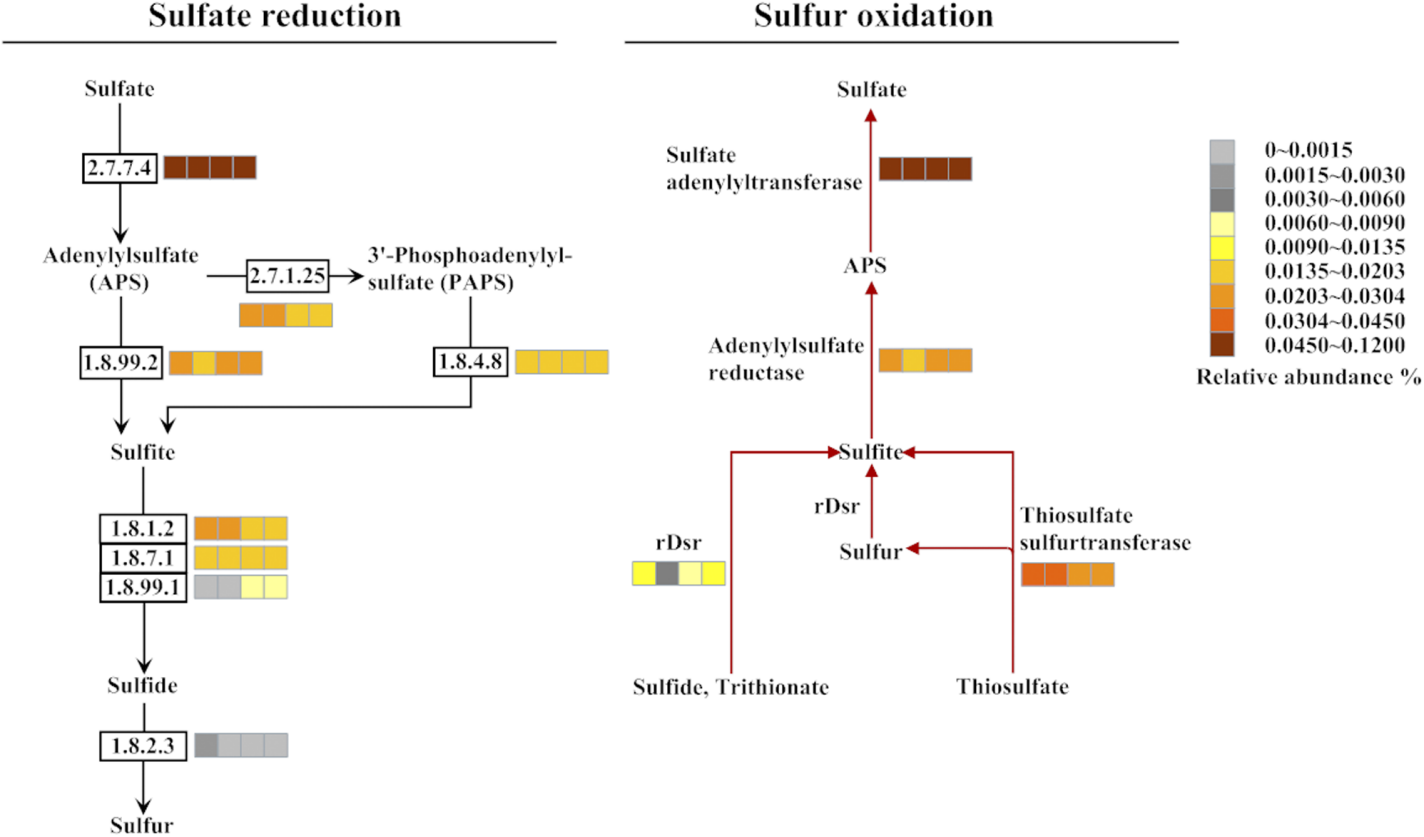
Relative abundances of the genes encoding enzymes involved in sulfate reduction and sulfur oxidation. Pathways are indicated by different colors. The EC numbers of enzymes are boxed. The relative abundances of the gene in the four samples are shown in the nearby color bar, in which the four segments from left to right represent PR1, PR4, DR7, and DR11. EC 2.7.7.4, sulfate adenylyltransferase; EC 2.7.1.25, adenylylsulfate kinase; EC 1.8.99.2, adenylylsulfate reductase; EC 1.8.4.8, phosphoadenylylsulfate reductase (thioredoxin); EC 1.8.1.2, assimilatory sulfite reductase (NADPH); EC 1.8.7.1, assimilatory sulfite reductase (ferredoxin); EC 1.8.99.1, sulfite reductase; EC 1.8.2.3, sulfide-cytochrome-c reductase (flavocytochrome c). rDsr, reverse dissimilatory sulfite reductase.

Potential pathways of sulfur oxidation and sulfate reduction were identified in the four metagenomes (Fig 5). The relative abundances of the genes responsible for reducing sulfate was in general high in the four datasets, excepting for sulfite reductase (EC 1.8.99.1) gene *dsrAB* and sulfide-cytochrome-c reductase (EC 1.8.2.3) gene *fccB* (Fig 5). In PR1, PR4, DR7, and DR11, the relative abundances of *dsrAB* were 0%, 0.0010%, 0.0067%, and 0.0083%, respectively, while the relative abundances of *fccB* were 0.0016%, 0.0006%, 0.0003%, and 0.0008%, respectively. Phylogenetic analysis showed that the DsrB sequence was close to that from *Thermodesulfovibrio*, *Syntrophobacterales*, *Desulfobacterales*, *Desulfatibacillum*, and Candidatus Rokubacteria (S7 Fig).

The reduced sulfur compounds were likely oxidized to sulfite by reverse dissimilatory sulfite reductase (rDsr, encoded by *rdsrAB*) and thiosulfate sulfurtransferase (Fig 5). Sulfite was subsequently converted through adenylylsulfate (APS) to sulfate. In the four datasets, *rdsrAB* was relatively more abundant in PR1 (0.0102%), DR7 (0.0090%), and DR11 (0.0113%) than in PR4 (0.0043%). Phylogenetic analysis showed that the sequence of rDsrB was related to uncharacterized taxa, some of which are affiliated to the order *Chlorobiales* and the genera *Rhodomicrobium* and *Thioalkalivibrio* (S7 Fig).

#### (iv) Genes related to hydrogenases

**Fig 6:**
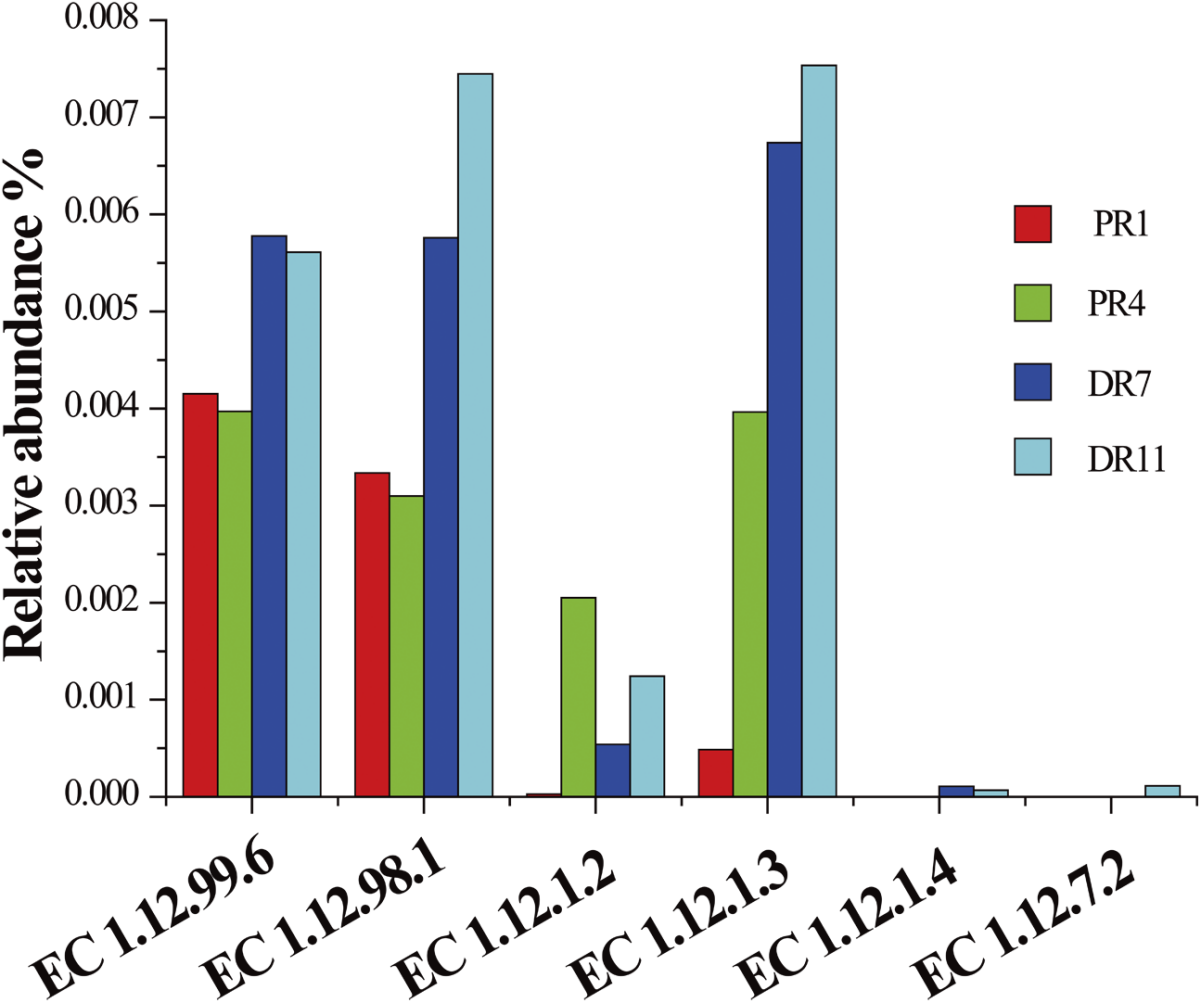
Relative abundances of hydrogenase genes in the four metagenomes. EC 1.12.99.6, hydrogenase or hydrogen:acceptor oxidoreductase; EC 1.12.98.1, F_420_-reducing hydrogenase; EC 1.12.1.2, NAD^+^-reducing hydrogenase; EC 1.12.1.3, NADP^+^-reducing hydrogenase; EC 1.12.1.4, bifurcating hydrogenase; EC 1.12.7.2, ferredoxin hydrogenase.

Hydrogen:acceptor oxidoreductase (or hydrogenase, EC 1.12.99.6), F_420_-reducing hydrogenase (EC 1.12.98.1), and NADP^+^-reducing hydrogenase (EC 1.12.1.3) were the main types of hydogenases in PR4, DR7, and DR11, while hydrogen:acceptor oxidoreductase and F_420_-reducing hydrogenase were the major hydrogenases in PR1 (Fig 6). In addition, NAD^+^-reducing hydrogenase (EC 1.12.1.2) was also present in the four samples but in relatively low abundances. These four hydrogenases belong to the [NiFe]-hydrogenase family. Genes related to [FeFe]-hydrogenase (bifurcating hydrogenase, EC 1.12.1.4) and [Fe]-hydrogenase (ferredoxin hydrogenase, EC 1.12.7.2) were only detected in DR7 and DR11 and in low abundance.

As revealed by phylogenetic analysis (S8 Fig), the large subunit of hydrogen:acceptor oxidoreductase (HyaB) was relatively closely related to those from the orders *Oceanospirillales*, *Chromatiales*, *Thiotrichales*, *Chlorobiales*, *Myxococcales*, *Desulfuromonadales*, *Desulfobacterales*, and *Clostridiales*; the beta subunit of F_420_-reducing hydrogenase (FrhB) was relatively closely related to those from the orders *Desulfobacterales*, Candidatus *Caldiarchaeum*, *Methanobacteriales*, *Methanosarcinales*, *Methanomicrobiales*, *Archaeoglobales*, *Methylococcales*, and *Rhizobiales* (S8 Fig).

## Discussion

### Microbial community structure

In this study, Illumina-based 16S amplicon sequencing was utilized to analyze the microbial diversity of four deep-sea sediments from the hydrothermal fields of Pacmanus and Desmos in Manus Basin. The taxonomic distribution of OTUs indicated that Proteobacteria and Thaumarchaeota were the most abundant bacterial phylum and archaeal phylum, respectively. Thaumarchaeota was relatively more abundant in the microbial communities of Pacmanus than in the microbial communities of Desmos, suggesting a possibly more important role of these archea in ammonia oxidization in the sediment of Pacmanus hydrothermal field. These results are consistent with the previous reports that Proteobacteria is the dominant community in marine environments and was found in deep-sea sediments and hydrothermal systems [18,37]. Likewise, Thaumarchaeota has been observed in the sediments of Southwest Indian Ridge and Okinwa Trough [18,38,39].

In Proteobacteria, *Gammaproteobacteria* and *Epsilonproteobacteria* were abundant in all four samples. *Gammaproteobacteria* has been widely detected in deep-sea environments [18,37,40–43], and *Epsilonproteobacteria* was reported to occur in the hydrothermal systems of Mid-Atlantic Ridge, Mariana Arc seamounts, and Okinawa Trough [44–46]. In our study, the presence of the genera *Sulfurovum*, *Sulfurimonas*, *Arcobacter*, and *Sulfurospirillum* within *Epsilonproteobacteria* suggested that these members potentially participated in sulfur oxidization and reduction as well as nitrate reduction. These observations are in line with the documented reports that many SOB and nitrate reducers belonging to these genera existed in hydrothermal systems [47–52]. Symbiotic and free-living sulfur oxidizers affiliated to *Cocleimonas* and *Thioprofundum* have been isolated from sand snail and vent chimney [53,54]. Sulfur-oxidizing gammaproteobacteria associated with these two genera were also detected in our datasets. The abundance of *Epsilon-* and *Gammaproteobacteria* indicated potential contributions of these bacteria to the oxidization of sulfur compounds in Pacmanus and Desmos fields. Chemoautotrophic *Epsilon-* and *Gammaproteobacteria* are primary producers using inorganic sulfur compounds as an energy source in deep-sea hydrothermal fields [55]. The total sulfur compound content was relatively higher in the sediments of Desmos, which is consistent with the observation that a higher abundance of SOB occurred in DR7 from Desmos. These sulfur compounds are likely originated from deposition of S^0^, H_2_S and SO_4_^2-^ contained in vent fluids or plumes [1]. There possibly exist different primary sulfur components in the sediments of Desmos and Pacmanus, as supported by the previous reports that Pacmanus hydrothermal deposits have polymetallic type of sulfide minerals, while Desmos hydrothermal deposits contain abundant native sulfur [1,2].

*Desulfobacterales* has been demonstrated as the dominant and active SfatRB in the Elkhorn Slough mat [56]. In our study, various members of *Desulfobacterales* were found in the four samples, suggesting a possible participation of these bacteria in sulfate reduction associated with anaerobic degradation of hydrocarbon in the sediments as reported previously [57]. Given the detection of TOC and TS in the sediments of our study, the existence of sulfur-oxidizing and sulfate-reducing bacteria in the microbial communities indicated potential contributions of these microbes to sulfur compounds and/or organic matter utilization and sulfur and carbon cycles. In addition, we observed in all samples striking abundances of *Nitrosopumilus*, *Nitrospira* and *Nitrospina*, which are ammonia-oxidizing archaea (AOA) and NOB, respectively [58–60]. The coexistence of these three genera suggested that different types of microbes participated in the conversion of ammonia to nitrate. Although there was no clear difference of TN content in the sediments, the ammonia-oxidizer *Nitrosopumilus* was relatively more abundant in Pacmanus than in Desmos. The richness of AOA and SOB hinted that ammonia and reduced sulfur compounds were potential energy sources fueling the microbial communities in these habitats.

### Metabolic profile–(i) Carbon metabolism

The metabolic profiles of the four samples were comparatively analyzed based on metagenomic sequencing. For carbon metabolism, we found that key enzymes participating in CBB cycle, WL pathway, and DC/4-HB or 3-HP/4-HB cycle existed in all four metagenomic datasets. RbcL, the key enzyme of CBB cycle, was related to that of *Nitrosococcus*, *Sedimenticola*/*Thiothrix*, and *Planctopirus*, which are known to be oxidizers of ammonia and sulfur [59,61–64]. WL-pathway has been reported to operate in diverse methanogens and sulfate reducers that fix CO_2_ in marine environments [36]. In our study, CdhA, the key enzyme of WL pathway, was homologous to that of Candidatus Bathyarchaeota and Candidatus Thorarchaeota, the former being a methanogen, and the latter being a sulfur reducer [65,66]. Since the *abfD* gene is highly abundant in all four metagenomes, DC/4-HB or 3-HP/4-HB cycle was likely one of the prevalent CO_2_ fixation pathways in our samples. AbfD-based phylogenetic analysis and 16S sequencing data indicated the existence of *Nitrosopumilus* and *Syntrophobacter*, which are known as ammonia oxidizer and sulfate reducer, respectively [58,67,68]. For the rTCA cycle, the key enzyme AclA was closely related to that from *Nitrospira*, which was also present in the 16S data, and *Thermoplasmatales*, which in nature are nitrite-oxidizing and methanogenic, respectively [59,60,69]. Taken together, all these results suggest that autotrophic CO_2_ fixation coupled to nitrification, sulfur metabolism, and methanogenesis may be present in the microbial communities of the hydrothermal fields of Pacmanus and Desmos. Genes associated with methane metabolism were identified in PR1, PR4, DR7 and DR11 in low abundances (0.75%, 0.79%, 0.89% and 0.86%, respectively), however, the methyl coenzyme M reductase gene, which functions in methanogenesis to produce methane and in the anaerobic oxidation of methane [70,71], was absent in all four metagenomic datasets, suggesting that methane may not be an important energy source for the local microbes.

### (ii) Nitrogen metabolism

Nitrification and denitrification are essential steps in the global nitrogen cycle [72]. In our study, an abundant key genes of these two processes were present in the metagenomes. In autotrophic ammonia oxidizers, AMO and HAO are two key enzymes necessary for energy conservation during ammonia oxidation [72]. In our study, both AmoA-based phylogenetic analysis and 16S rRNA gene analysis indicated that *Nitrosopumilus* and *Nitrosospira* were the primary ammonia oxidizers. The phylogenetic trees based on NarH and NapB showed that they were homologous to those from *Pseudomonas*, Candidatus *Methylomirabilis*, Candidatus Rokubacteria, *Nitrospira*, *Nitrospina*, and Candidatus *Scalindua*, members of which have been reported as nitrate reducer, nitrite oxidizer, and anammox bacteria in freshwater sediments and deep-sea hydrothermal sediments [59,60,73–76]. The genera of *Pseudomonas*, *Nitrospira*, and *Nitrospina* were also identified in the four 16S datasets. These results indicated a diversity of microorganisms responsible for utilization of ammonia and nitrate in the deep-sea sediments of Pacmanus and Desmos.

### (iii) Sulfur metabolism

Bacterial sulfate reduction and sulfur oxidation pathways were identified in the microbial communities of our study. The DsrB sequences in the four metagenomes were closely related to those from the class *Deltaproteobacteria* (77%-94% similarity), the genus *Thermodesulfovibrio* (70% similarity), and the phylum Candidatus Rokubacteria (~44% similarity), which have been identified as SfatRB in freshwater and deep-sea environments [56,76–79]. Sulfate in the sediments as an electron acceptor was likely utilized by potential SfatRB. A previous report predicted Candidatus Rokubacteria to be an important and unrecognized versatile player in sulfate reduction and nitrite oxidation in groundwater sediment [76]. It is noteworthy that we identified for the first time Candidatus Rokubacteria as a potential sulfate reducer in the deep-sea sediments of hydrothermal fields, which expands our knowledge of the taxonomic distribution of SfatRB in deep-sea hydrothermal systems. The reduced sulfur compounds in the sediments of Pacmanus and Desmos were likely oxidized to sulfate by rDsr via bacterial reverse sulfate reduction pathway, which is known to be present in the microbial communities of vent chimneys [80]. The phylogenetic closeness of rDsrB to the counterparts in *Thioalkalivibrio*, *Rhodomicrobium*, and *Chlorobiales* suggested that these microbes possibly played a role in sulfur oxidation in Pacmanus and Desmos hydrothermal fields, which is in line with the previous observations that these microbes occurred in hydrothermal chimneys and marine sediments [35,81,82].

### (iv) Hydrogen oxidation

Hydrogen as an electron donor can be reversibly oxidized by hydrogenase coupled to reduction of sulfate, Fe(III), and nitrate to produce energy for microorganisms [35,83,84]. Hydrogenases are classified into three families, i.e. [NiFe]-, [FeFe]-, and [Fe]-hydrogenases, based on the structure of active site [84]. In our study, [NiFe]-hydrogenase was more prevalent compared to the other types, as evidenced by a strikingly higher abundance of [NiFe]-hydrogenase gene in the four metagenomes, which is in agreement with the reports that [NiFe]-hydrogenase is the primary hydrogenase family in the microbial communities of hydrothermal plumes and vent chimneys [35,85]. Phylogenetic analysis revealed that HyaB and FrhB were likely derived from members of the sulfur oxidizing bacteria *Chromatiales*, *Thiotrichales*, and *Chlorobiales* [35,63,82], the sulfate reducing bacteria *Myxococcales*, *Desulfuromonadales*, *Desulfobacterales*, *Archaeoglobales*, and *Clostridiales* [56,86–89], the archaeal methanogens *Methanomicrobiales* and *Methanosarcinales* [69], and the bacterial methanotroph *Methylococcales* [90]. Hence, it appears that hydrogenases are widely distributed in bacteria and archaea. Members belonging to the orders of *Chromatiales*, *Myxococcales*, *Desulfuromonadales*, *Desulfobacterales*, and *Clostridiales* were also observed in our 16S sequencing datasets. It is possible that hydrogen as an energy source may be utilized by microbes in these sediments, and that this process is coupled to various metabolic processes including sulfur oxidation, sulfate reduction, and methane metabolism as observed in the vent plumes and chimneys from Guaymas Basin, Eastern Lau Spreading Center, and Southwest Indian Ridge [35,85,91].

## Conclusions

In this study, we conducted the first investigation on microbial diversity and potential metabolic profiles of the microbial communities inhabiting the sediments of the hydrothermal fields in Pacmanus and Desmos. We found that all microbial communities were dominated by Proteobacteria, and that ammonia- and sulfur-oxidizing prokaryotes likely played essential roles in the cycling of nitrogen and sulfur as well as in CO_2_ fixation. Ammonia, reduced sulfur compounds, and hydrogen as potential important energy sources were possibly utilized to fuel the microbial communities. These first observations add new insights into the metabolisms of the microbes in deep-sea hydrothermal fields.

## Supporting information

S1 Table 16S rRNA gene sequencing information and microbial diversity index. (DOC)

S2 Table General information of the four metagenomic datasets. (DOC)

S1 Fig Phylogenetic tree based on the amino acid sequences of RbcL. The tree was created with Neighbor-Joining method, using *Rhodococcus* species (ARE36203 and OQQ37473) as an outgroup. Bootstrap values are shown as percentages of 1000 bootstrap replicates. Sequences from the metagenomes of this study are indicated by red letters. Order and class are indicated in the right side. The scale bar represents 0.1 amino acid substitutions per site. α and γ represent the classes *Alphaproteobacteria* and *Gammaproteobacteria*, respectively.
(TIF)

S2 Fig Phylogenetic tree based on the amino acid sequences of the alpha subunit of Cdh. The tree was constructed with Neighbor-Joining method, using *Archaeoglobus fulgidus* (WP_048095537 and WP_010878596) as an outgroup. Bootstrap values are shown as percentages of 1000 bootstrap replicates. Sequences from the metagenomes of this study are indicated by red letters. Phylum is indicated in the right side. The scale bar represents 0.05 amino acid substitutions per site.
(TIF)

S3 Fig Phylogenetic tree based on the amino acid sequences of AclA. The tree was constructed with Neighbor-Joining method, using proteobacteria (SDR59450, OAD20009 and EGW53537) as an outgroup. Bootstrap values are shown as percentages of 1000 bootstrap replicates. Sequences from the metagenomes of this study are indicated by red letters. The scale bar represents 0.1 amino acid substitutions per site.
(TIF)

S4 Fig Phylogenetic tree based on the amino acid sequences of AbfD. The tree was constructed with Neighbor-Joining method, using Clostridiales species (WP_053954683 and WP_034425949) as outgroups. Bootstrap values are shown as percentages of 1000 bootstrap replicates. Sequences from the metagenomes of this study are indicated by red letters. Classes are indicated in the right side. The scale bar represents 0.05 amino acid substitutions per site.
(TIF)

S5 Fig **Phylogenetic tree based on the sequences of NarH (A) and NapB (B).** The trees were constructed with Neighbor-Joining method, using *Selenomonas* species and *Dechloromonas aromatica* as an outgroup in Figs A and B respectively. Bootstrap values are shown as percentages of 1000 bootstrap replicates. Sequences from the metagenomes of this study are indicated by red letters. Order and class are indicated in the right side. The scale bar represents 0.05 (A) or 0.1 (B) amino acid substitutions per site. α, β, γ, δ, and ε represent the classes *Alpha-*, *Beta-*, *Gamma-*, *Delta-*, and *Epsilonproteobacteria*, respectively.
(EPS)

S6 Fig Phylogenetic tree based on the sequences AmoA. The tree was constructed with Neighbor-Joining method, using gammaproteobacteria (AAW47734, AAB57809, and AAF03938) as an outgroup. Bootstrap values are shown as percentages of 1000 bootstrap replicates. Sequences from the metagenomes of this study are indicated by red letters. Genus and class are indicated in the right side. The scale bar represents 0.1 amino acid substitutions per site.
(TIF)

S7 Fig Phylogenetic tree based on the amino acid sequences of DsrB and the beta subunit of reverse dissimilatory sulfite reductase (rDsrB). The tree was constructed with Neighbor-Joining method, using *Pyrobaculum islandicum* (NC_008701) as an outgroup. Bootstrap values are shown as percentages of 1000 bootstrap replicates. Sequences from the metagenomes of this study are indicated by red letters. Genus/order and class are indicated in the right side. The scale bar represents 0.05 amino acid substitutions per site. α, γ, and δ represent the classes *Alpha-*, *Gamma-*, and *Deltaproteobacteria*, respectively.
(TIF)

S8 Fig **Phylogenetic tree based on the amino acid sequences of HyaB (A) and FrhB (B).** The trees were constructed with Neighbor-Joining method, using *Magnetospirillum* species (WP_011383527, CUW39305 and WP_068433304) and Actinobacteria (AKL72457 and CCM63982) as an outgroup in Figs A and B, respectively. Bootstrap values are shown as percentages of 1000 bootstrap replicates. Sequences from the metagenomes of this study are indicated by red letters; other sequences are from NCBI database with the accession numbers indicated. The scale bar represents 0.05 (A) or 0.1 (B) amino acid substitutions per site. Order and class are indicated in the Figs A and B. α, γ, and δ represent the classes *Alpha-*, *Gamma-*, and *Deltaproteobacteria*, respectively.
(EPS)
