# Supplementary material for: A comparative study revealed first insights into the diversity and metabolisms of the microbial communities in the sediments of Pacmanus and Desmos hydrothermal fields
Source: PLoS One. 2017 Jul 12;12(7):e0181048. doi: 10.1371/journal.pone.0181048 (PMC5507547; doi:10.1371/journal.pone.0181048)
Supplement: S1 Table — (DOC) [file pone.0181048.s001.doc]

**S1 Table. 16S rRNA gene sequencing information and microbial diversity index.**

|  | **PR1** | **PR4** | **DR7** | **DR11** |
| --- | --- | --- | --- | --- |
| **No. of raw paired-end reads** | 63071 | 74331 | 66522 | 76595 |
| **No. of effective tags** | 44107 | 49375 | 43088 | 49471 |
| **Percentage of effective tags (%)** | 69.9 | 66.4 | 64.8 | 64.6 |
| **Bases of effective tags (nt)** | 17925062 | 20401932 | 17927080 | 20527539 |
| **Average length of effective tags (nt)** | 406 | 413 | 416 | 415 |
| **No. of taxon tags** | 36865 | 32020 | 31504 | 28772 |
| **No. of OTUs** | 1169 | 1636 | 1710 | 1617 |
| **Chao1 index** | 1159.889 | 1615.943 | 1701.654 | 1607.072 |
| **Shannon index** | 7.198 | 7.876 | 8.265 | 7.926 |
