# Supplementary material for: A comparative study revealed first insights into the diversity and metabolisms of the microbial communities in the sediments of Pacmanus and Desmos hydrothermal fields
Source: PLoS One. 2017 Jul 12;12(7):e0181048. doi: 10.1371/journal.pone.0181048 (PMC5507547; doi:10.1371/journal.pone.0181048)
Supplement: S2 Table — (DOC) [file pone.0181048.s002.doc]

**S2 Table. General information of the four metagenomic datasets.**

|  | **PR1** | **PR4** | **DR7** | **DR11** |
| --- | --- | --- | --- | --- |
| **Raw reads (Mbp)** | 13540.14 | 13161.72 | 12587.78 | 13605.92 |
| **No. of raw reads** | 90267600 | 87744800 | 83918533 | 90706133 |
| **Clean reads (Mbp)** | 13518.00 | 13140.24 | 12562.26 | 13586.06 |
| **No. of reads after quality control** | 90120000 | 87601600 | 83748400 | 90573733 |
| **No. of scaftigs** | 338499 | 333177 | 352292 | 377374 |
| **Average length of scaftigs (bp)** | 808.97 | 851.58 | 800.73 | 821.81 |
| **Scaftig N50 length (bp)** | 774 | 813 | 764 | 784 |
| **Maximum length of scaftig (bp)** | 35667 | 74775 | 37317 | 91363 |
| **No. of predicted unique gene** | 502725 | 509062 | 518954 | 567297 |
