# Supplementary figures and images for: A comparative study revealed first insights into the diversity and metabolisms of the microbial communities in the sediments of Pacmanus and Desmos hydrothermal fields
Source: PLoS One. 2017 Jul 12;12(7):e0181048. doi: 10.1371/journal.pone.0181048 (PMC5507547; doi:10.1371/journal.pone.0181048)

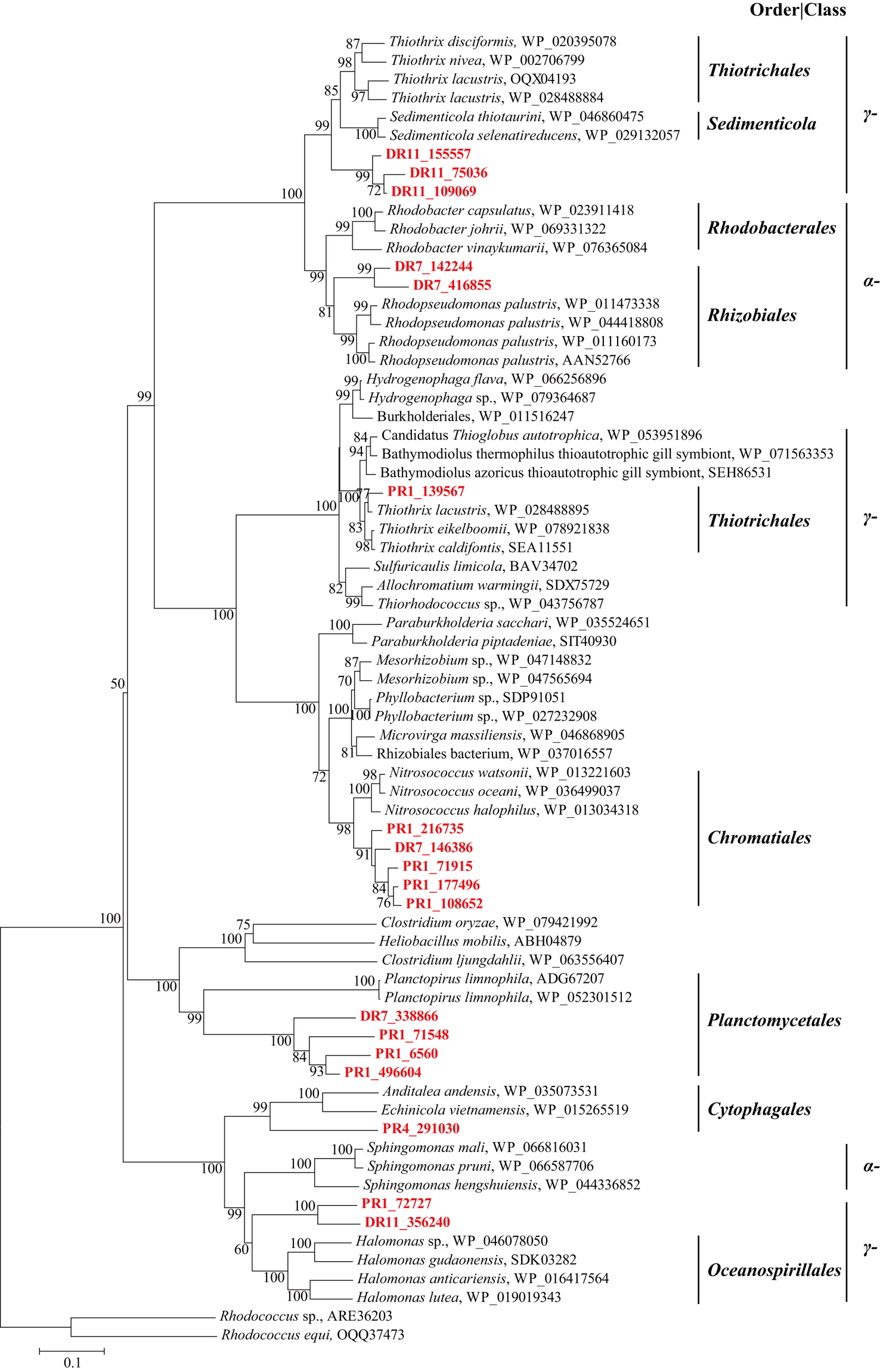

Supplement: S1 Fig — The tree was created with Neighbor-Joining method, using Rhodococcus species (ARE36203 and OQQ37473) as an outgroup. Bootstrap values are shown as percentages of 1000 bootstrap replicates. Sequences from the metagenomes of this study are indicated by red letters. Order and class are indicated in the right side. The scale bar represents 0.1 amino acid substitutions per site. α and γ represent the classes Alphaproteobacteria and Gammaproteobacteria, respectively. (TIF) [file pone.0181048.s003.tif]

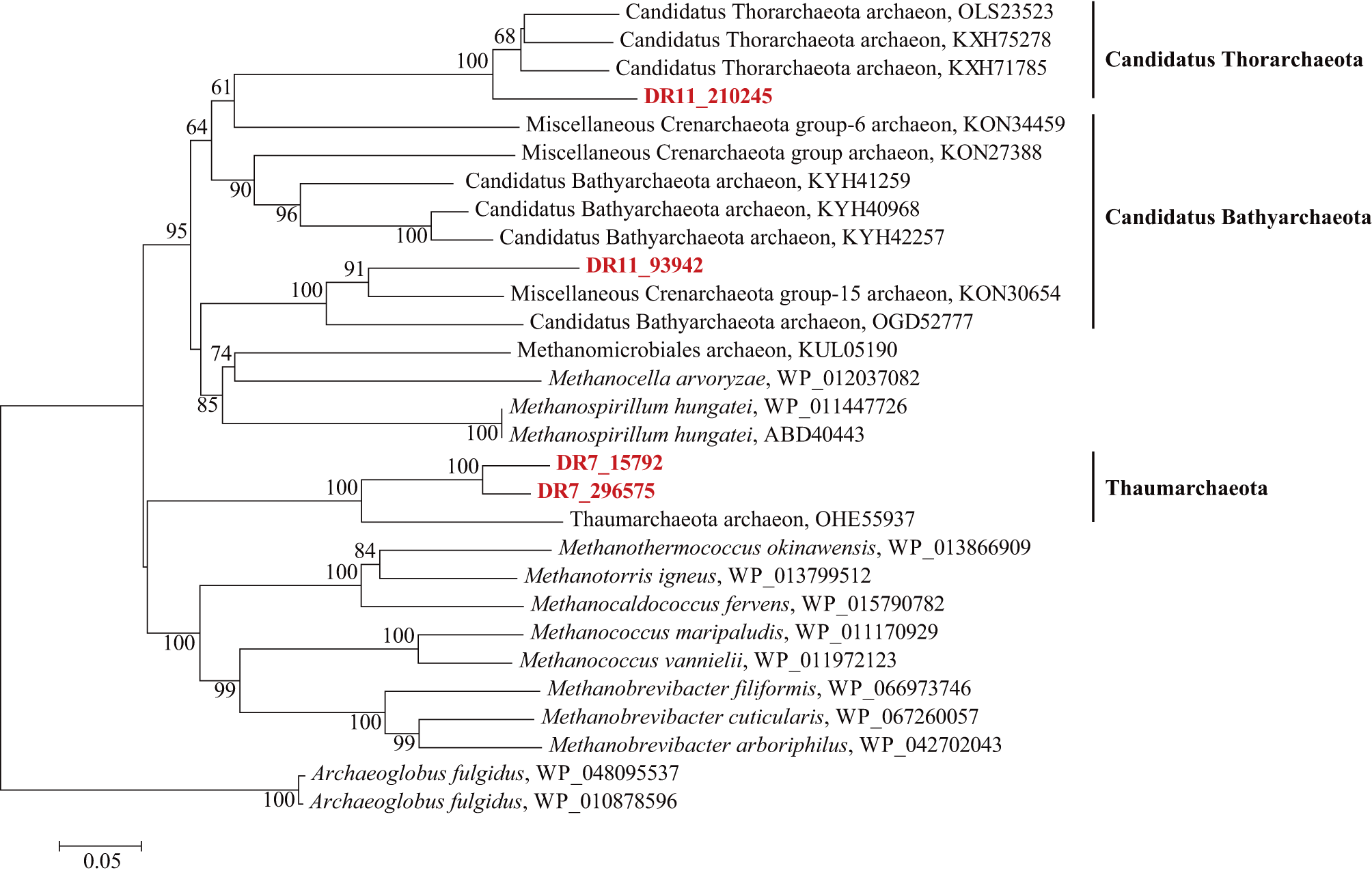

Supplement: S2 Fig — The tree was constructed with Neighbor-Joining method, using Archaeoglobus fulgidus (WP_048095537 and WP_010878596) as an outgroup. Bootstrap values are shown as percentages of 1000 bootstrap replicates. Sequences from the metagenomes of this study are indicated by red letters. Phylum is indicated in the right side. The scale bar represents 0.05 amino acid substitutions per site. (TIF) [file pone.0181048.s004.tif]

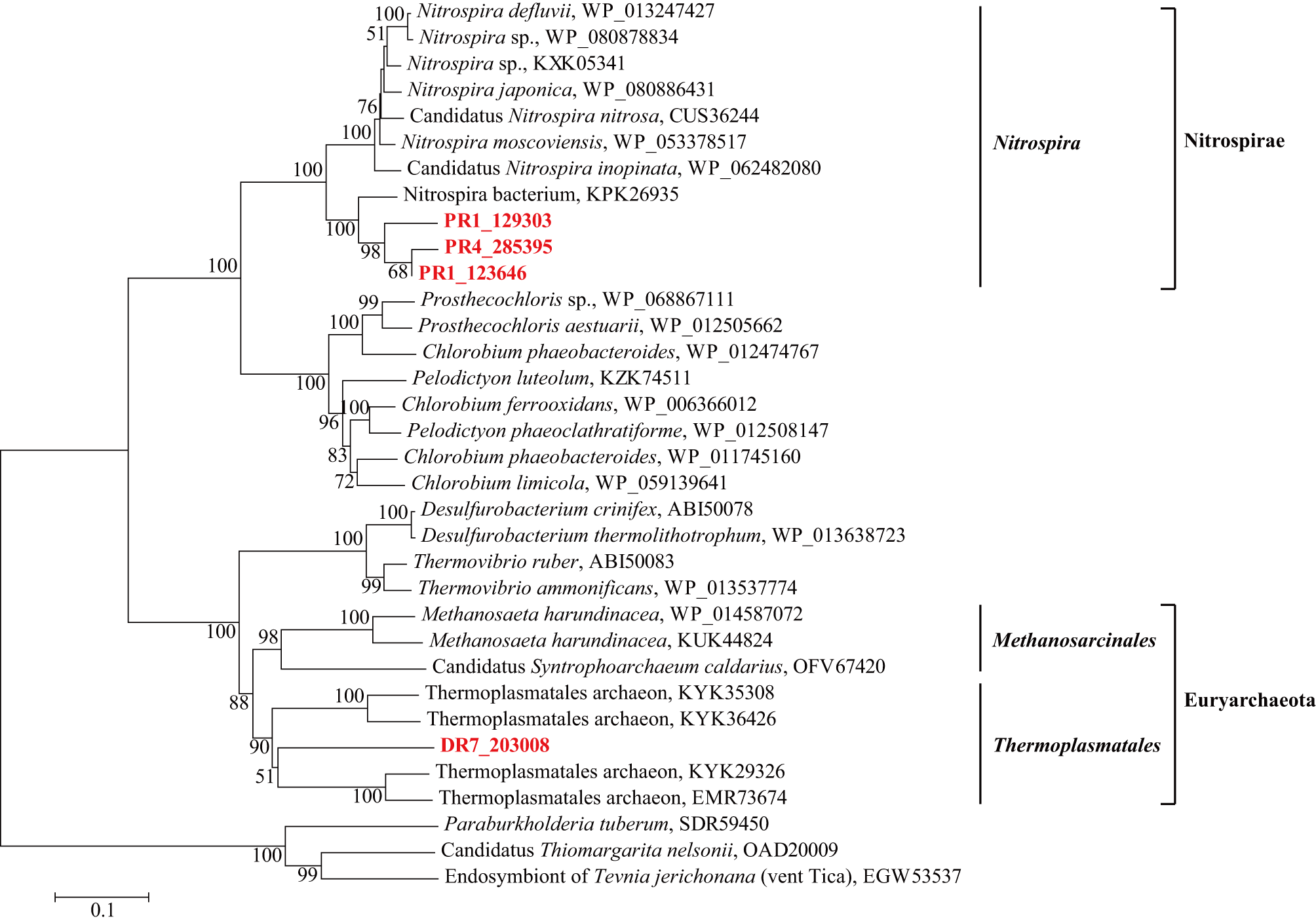

Supplement: S3 Fig — The tree was constructed with Neighbor-Joining method, using proteobacteria (SDR59450, OAD20009 and EGW53537) as an outgroup. Bootstrap values are shown as percentages of 1000 bootstrap replicates. Sequences from the metagenomes of this study are indicated by red letters. The scale bar represents 0.1 amino acid substitutions per site. (TIF) [file pone.0181048.s005.tif]

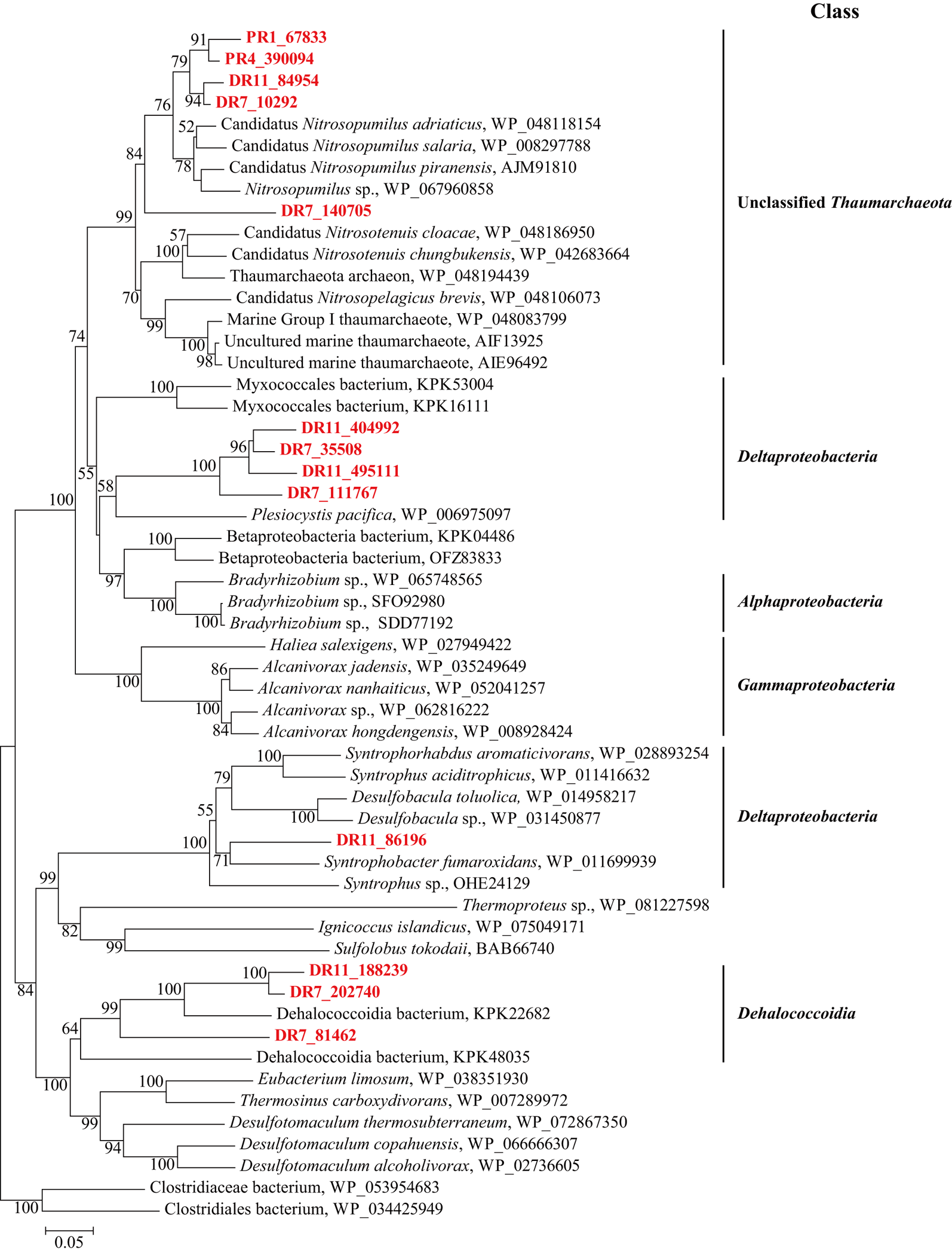

Supplement: S4 Fig — The tree was constructed with Neighbor-Joining method, using Clostridiales species (WP_053954683 and WP_034425949) as outgroups. Bootstrap values are shown as percentages of 1000 bootstrap replicates. Sequences from the metagenomes of this study are indicated by red letters. Classes are indicated in the right side. The scale bar represents 0.05 amino acid substitutions per site. (TIF) [file pone.0181048.s006.tif]

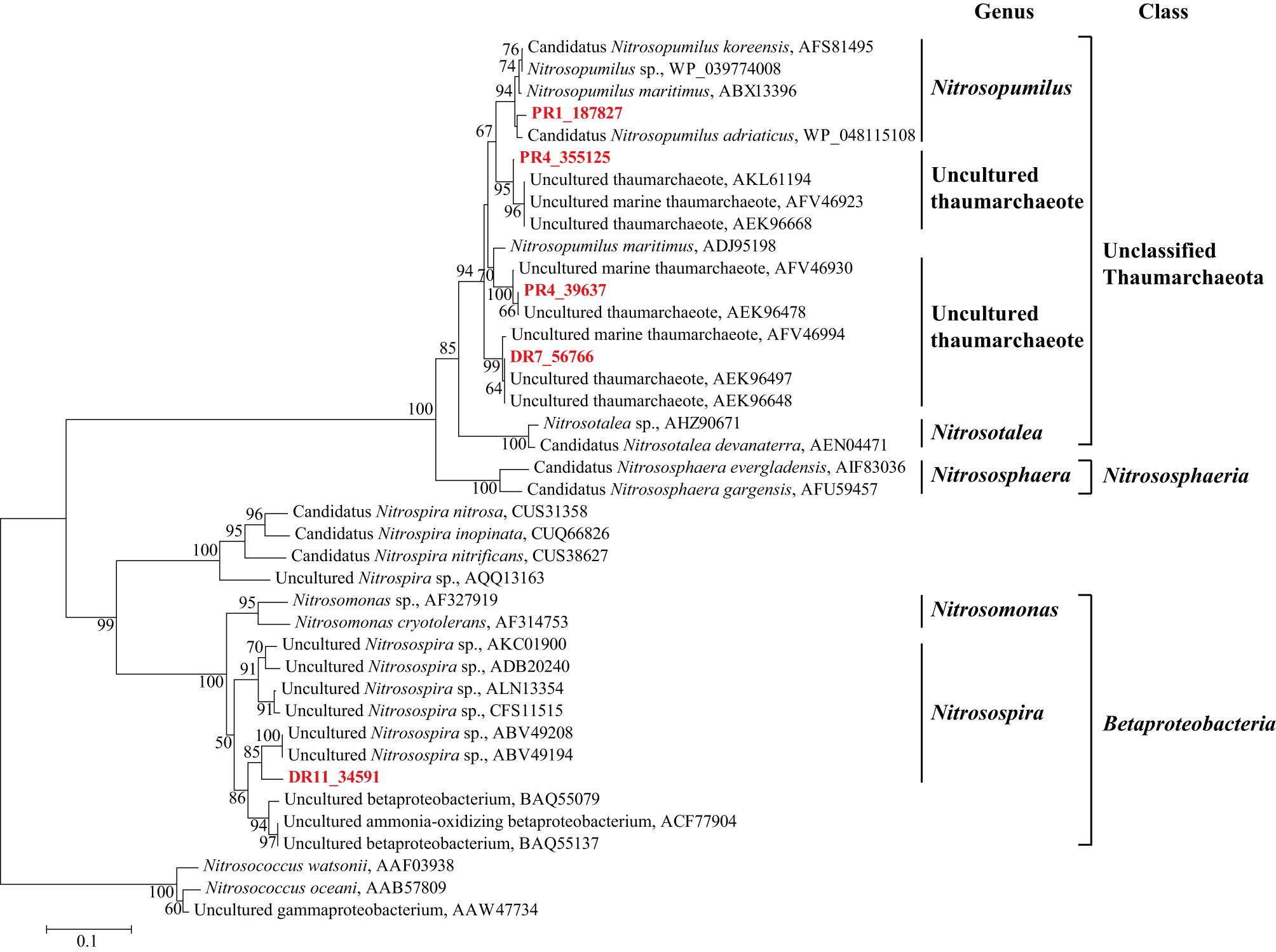

Supplement: S6 Fig — The tree was constructed with Neighbor-Joining method, using gammaproteobacteria (AAW47734, AAB57809, and AAF03938) as an outgroup. Bootstrap values are shown as percentages of 1000 bootstrap replicates. Sequences from the metagenomes of this study are indicated by red letters. Genus and class are indicated in the right side. The scale bar represents 0.1 amino acid substitutions per site. (TIF) [file pone.0181048.s008.tif]

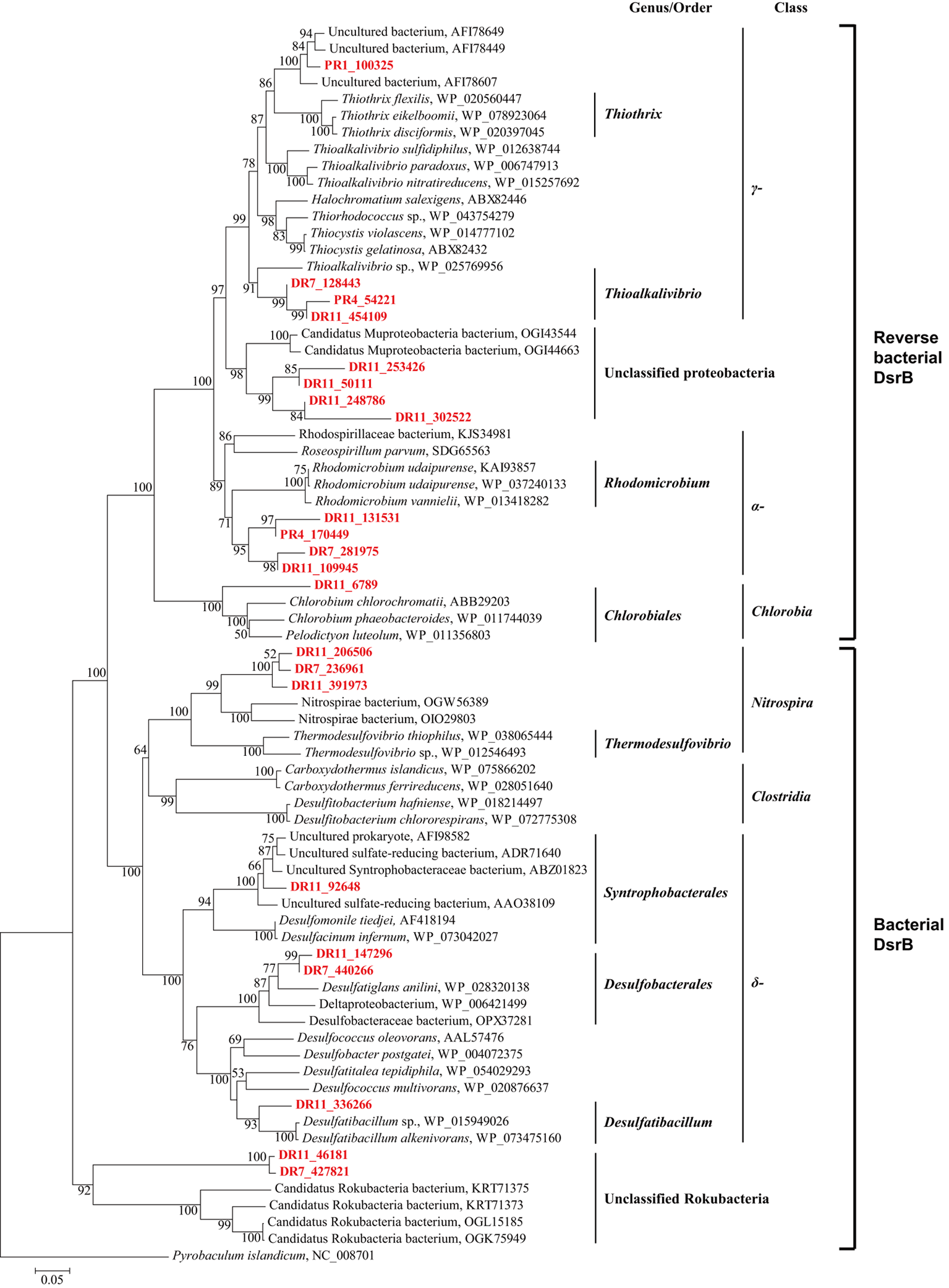

Supplement: S7 Fig — The tree was constructed with Neighbor-Joining method, using Pyrobaculum islandicum (NC_008701) as an outgroup. Bootstrap values are shown as percentages of 1000 bootstrap replicates. Sequences from the metagenomes of this study are indicated by red letters. Genus/order and class are indicated in the right side. The scale bar represents 0.05 amino acid substitutions per site. α, γ, and δ represent the classes Alpha-, Gamma-, and Deltaproteobacteria, respectively. (TIF) [file pone.0181048.s009.tif]
